# Genetics and environmental determinants of Alzheimer’s disease and related dementias in older Africans: a narrative review

**DOI:** 10.1186/s41021-025-00332-0

**Published:** 2025-10-09

**Authors:** Tobi Olajide, Oluwatimilehin Oladapo, Chukwuebuka Asogwa, Gideon Olajide, Ayomide Oyekan, Ayomide Fatola, Timileyin Olanrewaju, Damola Oyegbile, Ikechukwu Ugbo, Henry Oyoyo, Ridwan Kamarudeen, Olawale Famakin

**Affiliations:** 1 College Research and Innovation Hub, Ibadan, Nigeria; 2 College of Medicine, University of Ibadan, Ibadan, Nigeria

**Keywords:** Alzheimer’s disease, Dementia, Environmental risk factors, Genetics, Africa

## Abstract

The burden of Alzheimer’s disease and related dementias (ADRD) is rising in Africa, yet research remains limited compared to high-income regions. This narrative review investigated the genetic and environmental determinants of ADRD in older African populations, with a focus on the apolipoprotein E (APOE) gene. Although APOE ε4 is a strong risk factor for Alzheimer’s disease globally, its role in African populations appears less pronounced and variable, likely due to genetic diversity, evolutionary adaptations, and environmental interactions. We discussed the epidemiology of dementia in Africa, contrasting urban and rural patterns, and examined the distribution and effects of APOE alleles across African regions. Additionally, we reviewed how environmental exposures—including malaria, hypertension, HIV, heavy metals, pesticides, vitamin D deficiency, and air pollution—interact with APOE genotypes to influence cognitive decline. Critical challenges such as healthcare disparities, diagnostic inconsistencies, and underrepresentation in genomic studies were highlighted. Finally, we emphasized the need for longitudinal, multicenter research and initiatives like the African Dementia Consortium to bridge knowledge gaps and inform culturally tailored interventions for dementia prevention and care in Africa.

## Introduction

Africa’s aging population is experiencing rapid growth, signaling a significant demographic shift with substantial public health implications. Alzheimer’s Disease and Related Dementias (ADRD) are emerging as prominent health challenges, with the World Health Organization projecting a 204% increase in dementia cases by 2050, the majority of which will occur in low- and middle-income countries (LMICs) [1]. However, research on dementia has predominantly focused on high-income countries, leaving substantial gaps in understanding the burden of ADRD in African settings. Cognitive decline, a major precursor to dementia, is influenced by genetic, environmental, and healthcare factors that vary across regions. Alzheimer’s disease, which constitutes 60–80% of dementia cases globally, remains the most prevalent form of dementia; however, the distribution and prevalence of other forms of dementia in African countries remain inadequately studied [2].

Genetic factors, including the highly diverse genomic landscape of African populations, are pivotal in Alzheimer’s disease risk. However, African populations remain underrepresented in genomic research, thereby limiting insights into the genetic underpinnings of ADRD in these regions. The APOE gene is a central genetic determinant in late-onset Alzheimer’s disease [3]. APOE has three alleles: ε2, ε3, and ε4, with the ε4 allele being strongly associated with increased Alzheimer’s disease risk in European and North American cohorts. Conversely, the influence of APOE ε4 in African populations remains less well-defined and gives a mixed picture due to limited research in these groups. Emerging evidence suggests that the interaction between genetic diversity and environmental factors may shape the role of APOE in cognitive decline, highlighting the necessity for research that is specifically tailored to the unique socio-economic and environmental contexts of African countries.

This review explores the relationship between APOE genotypes and cognitive decline in African populations, emphasizing regional differences, the role of environmental and lifestyle factors, and the broader implications for dementia research and public health in Africa. 

## Epidemiology of dementia in Africa

The incidence and prevalence of dementia increase with age [4]. While Africa bears a significant proportion of the world’s aging population, there remains a paradoxical lower prevalence of dementia compared to other developed continents [5]. Several factors may contribute to this phenomenon, including lower life expectancy, underdiagnosis, suboptimal healthcare coverage for older individuals, and genetic diversity, among others.

Current estimates of dementia incidence in Sub-Saharan Africa (SSA) align with those in other low- and middle-income countries, with an incidence rate of 13.26 per 1,000 person-years, translating to approximately 367,698 new cases annually [6]. A systematic review by Mavrodaris et al. highlighted considerable variation in dementia prevalence, dependent on the criteria and methodology used in different studies [7]. Community-based studies employing diverse approaches and multiple rating scales have reported prevalence rates as high as 20%. Notably, geographical differences are evident: the lowest prevalence rates (around 2.3%) have been observed in Ibadan, Nigeria, and Al Kharga, Egypt. Western Africa generally reported lower prevalence (~ 3%), while regions such as Central, Eastern, and Southern Africa reported higher rates, exceeding 6%. Northern Africa presented intermediate prevalence figures, ranging from 2.3 to 5.1%.

Many African studies investigating the link between APOE ε4 and dementia employ varying diagnostic criteria, which significantly affect prevalence estimates and the interpretation of genetic associations. Commonly used frameworks include the Diagnostic and Statistical Manual of Mental Disorders (DSM-IV) and National Institute of Neurological and Communicative Disorders and Stroke/Alzheimer’s Disease and Related Disorders Association (NINCDS-ADRDA) criteria, which are considered gold standards in clinical research but may miss early or mild cases in African settings. Tools like the Community Screening Instrument for Dementia (CSI-D) and the 10/66 criteria offer culturally adapted alternatives that are more sensitive to subtle cognitive changes. For example, while a Tanzanian study reported a dementia prevalence of 6.4% using DSM-IV, the same population had a 21.6% prevalence under the 10/66 criteria [8]. Similarly, the EPIDEMCA study in Central Africa employed a two-stage diagnosis process using CSI-D followed by DSM-IV or NINCDS-ADRDA criteria, underscoring the diversity in diagnostic approaches [9].

### Urban vs. rural populations

Dementia prevalence is higher in urban areas compared to rural regions among older African adults. This disparity is primarily driven by urbanization, which increases cardiovascular risk factors such as hypertension, diabetes, and physical inactivity, all of which are associated with cognitive decline [5, 10]. Urban areas also benefit from better healthcare infrastructure, enabling earlier and more accurate dementia diagnoses, while rural areas face challenges due to limited diagnostic tools and healthcare personnel, leading to significant underdiagnosis [11].

In addition to healthcare access, social and environmental factors contribute to these disparities. Urban residents are more likely to experience higher levels of pollution and stress, both of which increase dementia risk. Conversely, rural populations may benefit from physical labor and traditional diets, which could offer some protective effects. However, limited healthcare access and lower health literacy in rural areas can delay diagnosis and reduce the effectiveness of treatment [12]. Key determinants of dementia prevalence include life expectancy and education. The longer life expectancy observed in urban areas is associated with a higher prevalence of dementia [13], while the generally lower life expectancy in rural regions may obscure the true burden of the disease. Higher education levels in urban populations are linked to enhanced cognitive reserve, which may reduce dementia risk by protecting against cognitive decline [10].

## APOE alleles

APOE protein is a critical cholesterol carrier protein involved in lipid transport and injury repair within the brain [7]. The APOE gene is characterized by three codominant alleles—ε2, ε3, and ε4—which give rise to six possible genotypes [14]. These alleles exhibit worldwide distribution frequencies of 8.4%, 77.9%, and 13.7%, respectively [15]. In sub-Saharan Africa, a study by Corbo and Scacchi reported a prevalence of 11.6%, 70.6%, and 17.8% for the APOE ε2, ε3, and ε4 alleles, respectively [16]. In Benin, the prevalence of these alleles was 10.3%, 74.2%, and 15.5%, respectively [16]. Nigeria, however, demonstrated a different distribution, with the ε2 allele occurring in 2.7%, ε3 in 67.7%, and ε4 in 29.6% of the population [16]. In East Africa, a comparative study in Kenya found that over 80% of the Nyeri Kikuyu population carried the APOE ε3/ε3 and ε3/ε4 genotypes, with the ε3/ε4 genotype being the most prevalent (48% versus 40% for ε3/ε3) [17]. In South Africa, Soo et al. reported a prevalence of 58.1% for ε3, 25.4% for ε4, and 16.5% for ε2 among rural older South Africans [18].

Genetically, the ε4 allele is well-established as the strongest risk factor for both early-onset and late-onset Alzheimer’s disease [19]. However, its association with Alzheimer’s disease is has a mixed picture in sub-Saharan African populations [20] and African-American populations [19] compared to Caucasian and Japanese populations [21]. While Kalaria found that the ε4 allele was present at two to three times higher frequency in Alzheimer’s disease patients than in the general healthy population [14], a subsequent study suggested that the elevated frequency of the APOE ε4 allele does not increase Alzheimer’s disease risk in sub-Saharan Africans [20]. These findings suggest that the ε4 allele contributes to dementia risk to a lesser extent in African populations than in Western cohorts. 

## Comparison of APOE genotype effects on dementia risk

### Western populations

Several studies have demonstrated that individuals of Caucasian descent carrying the APOE ε4 allele exhibit a significantly heightened susceptibility to Alzheimer’s disease compared to other ethnic groups, including African populations. For example, a study among British civil servants revealed a robust association between the APOE ε4 allele and an increased risk of dementia, both in heterozygous and homozygous carriers [22]. Similarly, a study examining the association between Alzheimer’s disease and APOE variants across different populations found a stronger correlation in East Asian and non-Hispanic White populations compared to non-Hispanic Black populations [23]. Additionally, another study found that Caucasians with the APOE ε4 allele were at a significantly higher risk for Alzheimer’s disease than African Americans and Hispanics [19]. These findings emphasize the stronger association between the APOE ε4 allele and Alzheimer’s disease in Western populations relative to other ethnic groups, including African populations.

### African populations

Numerous studies conducted across various African regions have identified APOE ε4 as a non-modifiable risk factor for dementia [5]. While studies in Western populations have firmly established the APOE ε4 allele as a significant risk factor for Alzheimer’s disease, the majority of studies within African populations have not demonstrated a similar association [5]. For instance, multiple studies conducted within the Nigerian population have consistently reported no significant association between the APOE ε4 allele and Alzheimer’s disease [24–26]. Likewise, studies in Kenyan, Beninese, and South African populations have also failed to establish a connection between the APOE ε4 allele and Alzheimer’s disease [17, 27, 28].

Despite the generally negative findings in African populations, some studies have identified a potential link between APOE ε4 and dementia risk. For example, a study conducted within the Tunisian population found an association between the APOE ε4 allele and an increased dementia risk [29]. Similarly, research involving African Americans and Yoruba Nigerians revealed notable differences in how the APOE ε4 allele impacts Alzheimer’s disease risk. Among African Americans, possessing one or two copies of the APOE ε4 allele significantly heightened the risk of developing Alzheimer’s disease. However, among Yoruba Nigerians, only individuals carrying two copies of the APOE ε4 allele (homozygous carriers) exhibited a significantly increased risk [25]. The APOE ε4 allele is also linked to Alzheimer’s disease in Northern African populations [5]. The effect of the APOE ε4 allele on Alzheimer’s disease risk appears less pronounced, albeit a mixed picture, in some African populations compared to Western populations. This observed regional variation may be attributed to genetic, environmental, and lifestyle factors that modulate the relationship between APOE ε4 and dementia risk [5]. These factors warrant further investigation.

### Genetic diversity in Africa

Africa is characterized by the highest level of genetic diversity among all continents, attributable to its low level of linkage disequilibrium and short haplotype blocks [18]. Increasing the inclusion of indigenous African populations in genomic studies holds the potential to uncover valuable insights into the genetic underpinnings of dementia-related phenotypes, as well as associated risk and protective factors. Despite this rich genetic diversity, fewer than 2% of genome-wide association studies (GWAS) have incorporated African data, leaving much of this diversity unexplored [18]. Consequently, there is limited knowledge regarding the genetic and epigenetic factors influencing dementia phenotypes in indigenous African populations.

The relationship between the APOE ε4 allele and Alzheimer’s disease in African populations has been inconsistent. While APOE ε4 is strongly associated with Alzheimer’s disease in many populations globally, its connection to the disease in individuals of African ancestry appears to be more complex. Intra-African genetic heterogeneity complicates uniform assessment of APOE’s effects. For example, a study by Hall et al. in the Nigerian Yoruba population found no strong association between APOE ε4 and Alzheimer’s disease [30]. However, a study conducted by Rassas and his team in the Tunisian population revealed a robust association between a high frequency of the APOE ε4 allele and Alzheimer’s disease, consistent with findings in other global populations [31]. These intra-African disparities may reflect unique evolutionary adaptations to local environments that modulate the expression of APOE-related risks.

Admixture between African and non-African populations introduces further complexity. African Americans, for example, exhibit mixed haplotypes of African and European origins at APOE-related loci, which may influence the pathogenicity of the gene. In a study, the APOE ε4-TOMM40’523-L haplotype was strongly associated with Alzheimer’s disease risk in Caucasian populations but showed a weaker association in African American populations [32]. Another study identified novel gene regions at the 17p13.2 locus associated with a lower risk of Alzheimer’s disease in African American populations [33]. These findings underscore the impact of genetic admixture on dementia risk and disease progression.

In some African populations, protective APOE variants have been identified, which appear to confer resilience against Alzheimer’s disease and cognitive decline. A study by Rajabli et al. reported that a genetic variant at locus 19q13.31 significantly reduced APOE ε4-associated Alzheimer’s disease risk [34]. Another study by Bertholim-Nasciben et al. identified an African-specific A allele at rs10423769, which reduces Alzheimer’s disease risk in APOE ε4 homozygotes by approximately 75% [35]. Furthermore, an intriguing study by Fujioka and colleagues suggested a possible relationship between the high frequencies of APOE ε4 alleles in Africa and a protective mechanism against malaria infection, particularly in malaria-endemic regions. This may reflect an evolutionary advantage conferred by the APOE ε4 allele, which could have been selected for due to its protective effect against malaria [36].

## Interactions between APOE and disease conditions

### Malaria

APOE ε4 is associated with Alzheimer’s disease (AD) but may have been preserved through evolutionary pressure due to its role in malaria protection. A study by Fujioka et al. [36] found that plasma from APOE ε4/4 carriers inhibited the growth of *Plasmodium falciparum*, the malaria parasite, while plasma from APOE 3/3 carriers did not. This protective effect, likely related to APOE’s binding to very low-density lipoproteins (VLDLs), may explain why the APOE ε4 allele persists in populations with high malaria prevalence. Similar to how hemoglobinopathies confer protection, the persistence of APOE ε4 may result from its adaptive role in areas endemic with malaria.

APOE has three main isoforms: APOE 2, APOE 3, and APOE 4, differing by single amino acid changes. APOE 4 is associated with Alzheimer’s disease but may have been preserved through evolutionary pressure due to its protective role against malaria. A study carried out in Sub-Saharan Africa revealed that plasma from APOE 4/4 carriers inhibits the growth of *Plasmodium falciparum*, the malaria parasite, unlike plasma from APOE 3/3 carriers [36]. In this study, nine different P. falciparum parasite lines from a variety of geographical locations were evaluated in this study: 3D7, 1905 (Papa New Guinea(PNG)), Dd2 (Indochina), 11B3 (Honduras), FCB (Columbia), FCR3 (Gambia), FAB6 (South Africa), G134 (Ghana), and ItG2 (Southeast Asia). 13 plasma samples (7 APOE 4/4, 6 APOE 3/3) from donors were investigated. Six out of seven plasma samples from APOE 4/4 donors inhibited the growth of P. falciparum. The one exception could be explained by the lower level of APOE 4/4 in plasma APOE 4/4 treatment led to parasite disintegration within intact RBC; parasite death was found at all stages of maturation. This result indicated that human APOE 4/4 plasma could be a factor mediating malaria parasite death. These changes were not observed in malaria growth for APOE 3. This protective effect likely results from APOE 4’s preferential binding to very low-density lipoproteins (VLDLs), altering lipid interactions critical to parasite growth. The findings suggest malaria may have driven the selection of APOE 4 in populations with high malaria prevalence, similar to how hemoglobinopathies confer protection. Further research is needed to explore how different APOE genotypes interact with various *Plasmodium* species, potentially leading to new malaria treatments. Although this study offers intriguing insights into the potential role of APOE isoforms in malaria, several limitations must be acknowledged to contextualize the findings. First, the absence of an intact immune system in in vitro experiments significantly limits the interpretation of results. The complex interactions between APOE isoforms and the host’s innate and adaptive immune responses cannot be fully replicated outside a living organism. As such, the immunological mechanisms by which APOE might influence malaria progression or severity remain speculative.

Secondly, the lack of physiological context in vitro is a significant drawback. Key organ systems such as the liver, bone marrow, and spleen—central to the pathophysiology of malaria—are not represented in cell culture models. Furthermore, important systemic dynamics, including lipid metabolism and the influence of the blood-brain barrier (especially relevant in cerebral malaria), are not captured. The study’s focus on the intraerythrocytic stages of *P. falciparum* also means that any potential protective effects of APOE4 during earlier stages of infection, such as sporozoite invasion or liver-stage development, remain unexplored. Additionally, in vitro systems fail to consider gene-environment interactions, including the influence of co-infections, diet, microbiome composition, and other modulating environmental factors that could shape APOE’s function in vivo. Finally, while APOE4 plasma appeared to inhibit malaria in vitro, it is unclear whether these concentrations reflect physiological levels or whether APOE4 must be localized to specific tissues or cell types—such as endothelial cells or macrophages—to exert its full anti-malarial effect. These limitations underscore the need for in vivo studies to validate and extend the current findings.

### Hypertension

Hypertension is a major dementia risk factor, especially in Central and West Africa. Studies show that individuals with hypertension and the APOE ε4 allele face a higher dementia risk. The Atherosclerosis Risk in Communities study, which tracked dementia incidence from 1987 to 2017, found that poor cardiovascular health was associated with higher dementia risk. However, the study also indicated that APOE ε4 status did not significantly interact with cardiovascular health in influencing dementia risk. Interestingly, better cardiovascular health correlated with a lower risk of dementia across all APOE genotypes.

### Human Immunodeficiency Virus (HIV)

HIV-associated neurocognitive disorders (HAND), which range from mild impairments to severe dementia, are driven by chronic inflammation and immune activation caused by HIV. A study by Hoare and team in South Africa showed that young HIV-infected adults (≤ 35 years) with at least one APOE ε4 allele performed worse in verbal recall tests and exhibited corpus callosum atrophy compared to noncarriers. This allele dose effect suggests that APOE ε4 carriers might be more vulnerable to HIV-related neurodegeneration. While findings on the interaction between APOE ε4 and HIV are inconclusive, some studies suggest APOE ε4 may alter neuronal gene expression, impairing reparative functions and worsening the neurodegenerative process in HIV-infected individuals.

## Gene-environment interactions

### Educational attainment

Higher education is associated with a cognitive reserve that may delay dementia onset. Studies across low- and middle-income countries (LMICs) indicate that lower education levels correlate with higher dementia risk. In a case-control study conducted by Yusuf et al. in Nigeria, it compared older adults with vs. without type 2 diabetes mellitus (T2DM) and it was found that low education was strongly associated with dementia [37]. Similarly, observational cohort studies conducted in South Africa and Tanzania emphasize the protective role of education in mitigating dementia risk [8, 38]. However, all of these associations do not imply a causal effect on dementia prevalence. Education provides cognitive resilience, which may help individuals better cope with neuropathological changes associated with aging and dementia.

### Pesticides and heavy metals exposure

Environmental exposure to heavy metals and pesticides can interact with APOE alleles, increasing dementia risk. Metals like copper, zinc, and iron contribute to neurotoxicity by accumulating in amyloid plaques and disrupting metal homeostasis. In a study conducted by Bakulski et al. to review the association between lead, cadmium and manganese on Alzheimer’s disease and related dementias [39]. It showed Lead exposure may be associated with multiple neurodegenerative process and may not be specific to AD or dementia. Lead is a potent neurotoxicant capable of causing non specific brain disruption, resulting in oxidative stress, endoplasmic reticulum stress, mitochondrial damage, excitotoxicity, altered homeostatic metal signaling, inflammation and ultimately neuronal apoptosis. In animal models, lead treatment causes AD-related pathology including changes in AbetaPP (Amyloid beta protein precursor), Abeta (Amyloid beta), and tau, as well as memory deficits. In older adults, lead exposure is associated with lower cognitive status and longitudinal declines in cognition. In order to assess lead exposure risk in AD specifically, prospective evidence in human clinical samples is needed.

Cadmium exposure is primary through diet and cigarette sources, inhaled cadmium can enter through the olfactory bulb or the blood cerebrospinal fluid barrier into the brain, In studies on animal model, it causes oxidative stress, neuroinflammation and neuronal apoptosis. It induces neurotoxicity by changing the permeability of blood brain barrier, causing amyloid-beta aggregation and producing tau neurofibrillary tangles. In human aging studies, cadmium may be associated with decreased cognitive function and clinical AD specifically [39].

Exposure to pesticides like DDT has been linked to memory loss and cognitive decline, with studies showing that DDT elevates amyloid precursor protein levels, impairing amyloid-beta peptide clearance. Individuals with the APOE ε4 allele may be more susceptible to these environmental toxins, heightening their risk of Alzheimer’s disease. In sub-Saharan Africa, widespread use of organophosphate pesticides and unsafe mining practices has led to rising environmental exposure to neurotoxic agents, even in rural populations. For instance, a Nigerian study by Anetor and team reported a correlation between blood lead levels and markers of oxidative DNA damage in rural farmers, a population at risk of neurocognitive decline [40]. Another study conducted in South Africa linked pesticide exposure among agricultural workers to deficits in memory and executive function, raising concerns about long-term cognitive outcomes in genetically predisposed groups such as APOE ε4 carriers [41].

### Sunlight and vitamin D

Vitamin D deficiency is increasingly recognized as a risk factor for dementia, including Alzheimer’s disease. An experimental study by Mokry and colleagues supports a causal role for vitamin D in dementia risk, highlighting the importance of maintaining adequate vitamin D levels [42]. A systematic review by Sommer et al. also found that individuals with severe vitamin D deficiency had a significantly higher dementia risk compared to those with sufficient levels [43]. The study also found that low vitamin D levels were associated with an increased risk of Alzheimer’s disease. Vitamin D deficiency may contribute to neurodegenerative diseases by reducing inflammation and offering neuroprotection.

In-vitro studies have shown that Vitamin D enhances the clearance of amyloid plaques by macrophages, inhibit amyloid-induced neurotoxicity and apoptosis, display anti-oxidant properties, affect the metabolism of many neurotransmitters and regulates the expression of neurotrophic factors [44]. In a study conducted by Huebbe et al. The APOE4 allele is correlated with higher serum vitamin D levels compared with the APOE2 and APOE3 alleles, and healthy humans with the APOE4 allele display increased circulating vitamin D levels compared with noncarriers [45]. Hence, it seems APOE4 allele status may affect the vitamin D levels in AD patients, although the age at the onset of disease could also influence this relationship.

### Air pollution

Air pollution is a growing environmental concern, particularly in urban areas across Africa. Exposure to pollutants like particulate matter (PM2.5) and nitrogen dioxide (NO2) has been linked to increased dementia risk, as air pollution exacerbates existing health conditions such as hypertension, diabetes, and stroke. Studies in Latin America and other regions indicate that air pollution accelerates cognitive decline by activating inflammatory pathways [46]. In an African pollution study by Yashoun et al. exposure to pollutants like PM2.5 may contribute to the rising incidence of dementia, particularly in urban populations where pollution levels are highest [47].

The study by Cacciottolo et al. investigates how exposure to air pollution interacts with the APOE ε4 allele to influence the risk of cognitive decline and dementia [48]. In a cohort of older women, residing in areas with elevated PM2.5 levels significantly increased the risk of global cognitive decline and all-cause dementia, with these risks being especially pronounced among APOE ε4/4 carriers [5]. Experimental studies in transgenic mice expressing human APOE alleles further supported these findings, showing that exposure to nanosized particulate matter (nPM) elevated cerebral β-amyloid levels and induced neurodegenerative changes, particularly in APOE ε4 carriers [5]. These results suggest that APOE ε4 enhances susceptibility to air pollution-induced neurotoxicity, thereby increasing dementia risk [5]. More studies are needed to dig deeper into the complex interaction between environmental exposure and the APOE ε4 allele in the development of AD.

## Challenges

The healthcare access disparities, diagnostic inconsistencies, and lack of research infrastructure in Africa hinder the understanding and management of dementia. While Western nations benefit from advanced diagnostic tools, healthcare disparities, particularly for minority groups like African Americans, contribute to delayed diagnoses. In Africa, these challenges are even more pronounced, exacerbated by poor healthcare infrastructure, especially in rural areas, and cultural perceptions that dementia is simply a normal part of aging. As a result, diagnoses are often missed or delayed, particularly for high-risk APOE ε4 carriers. Furthermore, the absence of standardized and culturally relevant diagnostic tools further complicates accurate assessments of dementia. Tools developed in Western settings fail to account for local linguistic, educational, and cultural variations, leading to potential misdiagnoses or missed diagnoses in African populations.

Moreover, research gaps, particularly in large-scale, longitudinal studies, limit the knowledge of how APOE genotypes affect dementia in African populations. This lack of research hinders the development of targeted interventions for APOE ε4 carriers, who are especially vulnerable to environmental and genetic factors that exacerbate dementia risk.

## Future directions

To address the ongoing challenges, several interventions are necessary to improve dementia diagnosis, care, and understanding in African populations. These include:

### Longitudinal studies

Long-term studies are crucial to understanding the role of the APOE gene in dementia risk and cognitive aging. Sub-Saharan Africa’s aging population is expected to rise significantly by 2050, emphasizing the need for targeted research to track APOE’s influence on cognitive decline over time. Such studies will provide essential insights into genetic predispositions and help tailor public health strategies specifically for African populations.

### Multicenter and regional studies

Africa, with its vast genetic diversity, requires more comprehensive, multicenter studies to capture the full range of genetic, environmental, and sociocultural factors that influence dementia risk. With less than 2% of global genome-wide association studies (GWAS) focusing on African populations, there is a critical need for more research that reflects this diversity. The Human Health and Heredity in Africa (H3Africa) study has already uncovered over 3 million previously unrecognized genetic variants, suggesting the immense untapped potential for African-centered genetic research. Such efforts could greatly advance the understanding of brain aging, Alzheimer’s disease, and cognitive decline in Africa.

### The African Dementia Consortium (AfDC)

The African Dementia Consortium is working to advance dementia research on the continent by building clinical and socioeconomic datasets, expanding biobanking efforts, and conducting research in key areas like genetics and biomarkers. The consortium, which includes over 100 researchers from across Africa, plays a pivotal role in addressing the continent’s dementia research needs. Collaborative initiatives like the Recruitment and Retention for Alzheimer’s Disease Diversity Cohorts (READD-ADSP) further enhance these efforts by aiming to recruit 5,000 Africans to explore the genetic aspects of Alzheimer’s disease. Intensification of efforts by this consortium will go a long way in advancing dementia care and research across Africa.

## Conclusion

This study emphasizes the complex interplay of genetic, environmental, and sociocultural factors shaping dementia risk in African populations, particularly the mixed picture of the APOE ε4 allele. Although APOE ε4 is a well-established genetic risk factor for Alzheimer’s disease globally, its less pronounced and variable effect in African populations suggests the influence of unique evolutionary adaptations and diverse genetic backgrounds. However, this does not negate its relevance; rather, it highlights the importance of context-specific research that accounts for gene-environment interactions, such as those involving exposure to heavy metals, pesticides, and other environmental toxins.

These insights carry substantial public health and policy implications. First, the less pronounced APOE ε4 effect calls for the development of culturally and genetically tailored risk assessment tools for dementia in African settings, moving away from one-size-fits-all models derived from non-African populations. Second, the identification of modifiable environmental risk factors points to opportunities for public health interventions—such as regulatory policies to reduce environmental neurotoxins, occupational safety standards, and awareness campaigns to promote cognitive health.

Healthcare systems in Africa must prioritize early detection and prevention strategies that are both feasible and sustainable. This includes integrating dementia risk screening into routine geriatric care, investing in community-based health education, and training primary healthcare workers in basic cognitive assessment. Moreover, policies that promote brain health across the lifespan—such as those enhancing nutrition, education, and cardiovascular health—can yield long-term dividends in dementia prevention.

Ultimately, addressing the challenges of limited diagnostic infrastructure and research capacity requires sustained investment and international collaboration. Strengthening local biobanking, genomics, and neuroepidemiological research is critical to uncovering context-specific drivers of dementia and tailoring interventions accordingly. By translating these findings into concrete policies and programs, we can improve dementia care across the continent and contribute meaningfully to the global effort to combat neurodegenerative diseases.

## Data Availability

No datasets were generated or analysed during the current study.

## Abbreviations

AD: Alzheimer’s Disease
ADRD: Alzheimer’s Disease and Related Dementias
AfDC: African Dementia Consortium
APOE: Apolipoprotein E
CSI-D: Community Screening Instrument for Dementia
CSF: Cerebrospinal Fluid
DDE: Dichlorodiphenyldichloroethylene
DDT: Dichlorodiphenyltrichloroethane
DSM-IV: Diagnostic and Statistical Manual of Mental Disorders
GWAS: Genome-Wide Association Studies
H3Africa: Human Health and Heredity in Africa
HAND: HIV-Associated Neurocognitive Disorders
HIV: Human Immunodeficiency Virus
LMIC: Low- and Middle-Income Country
NINCDS-ADRDA: National Institute of Neurological and Communicative Disorders and Stroke/Alzheimer’s Disease and Related Disorders Association
RBC: Red Blood Cell
READD-ADSP: Recruitment and Retention for Alzheimer’s Disease Diversity Cohorts in the Alzheimer’s Disease Sequencing Project
SSA: Sub-Saharan Africa
VLDL: Very Low Density Lipoprotein
WHO: World Health Organization
